# Complex Size and Surface Charge Determine Nucleic Acid Transfer by Fusogenic Liposomes

**DOI:** 10.3390/ijms21062244

**Published:** 2020-03-24

**Authors:** Marco Hoffmann, Nils Hersch, Sven Gerlach, Georg Dreissen, Ronald Springer, Rudolf Merkel, Agnes Csiszár, Bernd Hoffmann

**Affiliations:** Forschungszentrum Jülich, Institute of Biological Information Processing; IBI-2, 52428 Jülich, Germany; mar.hoffmann@fz-juelich.de (M.H.); n.hersch@fz-juelich.de (N.H.); s.gerlach@fz-juelich.de (S.G.); g.dreissen@fz-juelich.de (G.D.); r.springer@fz-juelich.de (R.S.); r.merkel@fz-juelich.de (R.M.); a.csiszar@fz-juelich.de (A.C.)

**Keywords:** fusogenic liposomes, transfection, nucleic acid complexation, membrane fusion

## Abstract

Highly efficient, biocompatible, and fast nucleic acid delivery methods are essential for biomedical applications and research. At present, two main strategies are used to this end. In non-viral transfection liposome- or polymer-based formulations are used to transfer cargo into cells via endocytosis, whereas viral carriers enable direct nucleic acid delivery into the cell cytoplasm. Here, we introduce a new generation of liposomes for nucleic acid delivery, which immediately fuse with the cellular plasma membrane upon contact to transfer the functional nucleic acid directly into the cell cytoplasm. For maximum fusion efficiency combined with high cargo transfer, nucleic acids had to be complexed and partially neutralized before incorporation into fusogenic liposomes. Among the various neutralization agents tested, small, linear, and positively charged polymers yielded the best complex properties. Systematic variation of liposomal composition and nucleic acid complexation identified surface charge as well as particle size as essential parameters for cargo-liposome interaction and subsequent fusion induction. Optimized protocols were tested for the efficient transfer of different kinds of nucleic acids like plasmid DNA, messenger RNA, and short-interfering RNA into various mammalian cells in culture and into primary tissues.

## 1. Introduction

During the last decades, numerous nucleic acid transfer methods for in vitro as well as in vivo purposes have been reported. However, a universally applicable transfection strategy that combines high bioavailability, low toxicity, and highest transfer efficiency has yet to be developed. In general, there are two main routes toward transfection: the chemical route based on nanoparticles or liposomal- and polymer-based formulations [1,2,3,4,5], and the viral route, where the viral envelope serves as carrier vehicle [6,7].

Most frequently used for in vitro applications are liposomal or polymer formulations due to easy handling. The classical liposomal formulation containing an equimolar mixture of a neutral and a positively charged lipid, e.g., DOPE/DOTAP, had already been introduced in the 1980s. However, in these lipofection methods, all nucleic acid transfer complexes are taken up via endocytosis [8,9,10] and are therefore destined to undergo lysosomal degradation [10,11]. To bypass this undesired process, so-called multi-component lipoplexes have been developed. Compared to the original system, they show 10 to 100 times higher DNA transfer efficiencies and use strategies like endosomal destabilization [11,12,13,14] or pH-adaptation using polymers with proton sponge buffering properties [15]. Additionally, nucleic acid protection strategies also become more and more popular. In this respect, co-incubation of liposome/DNA complexes with albumin, chitosan, or protamine not only reduced degradation but also enhanced DNA transfer efficiency [14,16,17,18,19]. Especially for in vivo applications, various types of nanoparticles have also been developed to stabilize and protect nucleic acids and to facilitate cell type-specific targeting [20].

Common drawbacks of liposome- or polymer-based transfection methods are sometimes low transfer rates [21,22] combined with significant cytotoxicity [23,24]. Both limitations are caused by the underlying uptake pathway via endocytosis. Beside pronounced variations in endocytic activity between different cell types and during the cell cycle [25], on their way to degradation, endosome encapsulated nucleic acids are prone to be targeted by pattern recognition receptors. These receptor types are highly enriched in endosomal structures. As part of cellular protection systems against viruses and bacteria, they initiate inflammatory pathways [26,27,28] and, ultimately, apoptosis [29]. To bypass such endosome-related protection mechanisms, various viruses transfer their nucleic acid directly into the cytoplasm via receptor-induced fusion with the plasma membrane of mammalian cells. Commercially available viral transfer systems are typically highly efficient. However, most systems go along with increased biosafety requirements, high prices, and limitations in nucleic acid sizes that can be packed into the viral particles [30,31].

Recently, we developed liposomal formulations with fusogenic capacity. These particles, called fusogenic liposomes (FLs), are formed from a mixture of neutral and cationic lipids with a small admixture of lipid analogs that contain a delocalized π-electron system [32]. They fuse with the plasma membrane of living cells with extraordinarily high efficiency without the need for viral peptides or other fusion-inducing proteins [33,34]. Such FLs were already used to transfer various biological macromolecules like lipids [33], proteins [35], and polyphenols [32] as well as synthetic beads [36]. Depending on their chemical characteristics, cargos are either transported directly into the cell cytoplasm [35] or intercalated into the plasma membrane [37].

Since electrostatic interactions between the positively charged FLs and the negatively charged cell surface support fusion, cargos that neutralize liposomal charge hamper fusion. This is because negatively charged cargos will efficiently interact with fusogenic liposomes and thereby reduce attractive interactions of the liposomes to the cell surface. On the other hand, positively charged cargos might barely interact with the liposomal formulation, making their transfer largely inefficient. Indeed, we found that direct incubation of FLs and negatively charged nucleic acids blocked fusion with mammalian plasma membranes. To eliminate this effect, we systematically varied complexation conditions to partially neutralize nucleic acids. Various protein and polymer-based neutralization reagents (NRs) have been analyzed, and optimal ratios of such reagents with every type of nucleic acids (NA, specifically, DNA, mRNA, siRNA) were identified. By further systematic variation of liposome composition, we ultimately identified optimum conditions for membrane fusion and thus the direct transfer of nucleic acids into the cell cytoplasm. Parallel analyses of the system optimized here, which was then named Fuse-It-mRNA, on cell toxicity and potential uptake routes could clearly show unaffected fusogeneity and nucleic acid transfer efficiency in the presence of endosomal blockers and proved high biocompatibility in short-term and long-term cell functional analyses [38].

## 2. Results

### 2.1. Neutralization of Nucleic Acids Is Essential for Their Efficient Transfer by Fusogenic Liposomes

Fusogenic liposomes (FLs) preferentially transfer neutral and negatively charged molecules into mammalian cells [32,33,35,37,39]. The high positive charge of FLs is hereby not only supporting the interaction of these molecules but is also responsible for the interaction of cargo loaded FLs with the slightly negatively charged surface of plasma membranes [40]. However, direct incubation of FLs with varying concentrations of nucleic acids (NAs) always resulted in only low transfer efficiencies, although positively charged lipids were described as neutralization reagents (NR) for NA to support NA transfer into mammalian cells [2,41,42]. We tentatively traced this finding to charge neutralization of the positively charged FL particles (DOPE/DOTAP/DiR, molar ratio 1/1/0.1) by their negatively charged cargo (Figure S1). Therefore we tested the pre-incubation of NAs with positively charged molecules to enhance transfer efficiency. 

**Figure 1:**
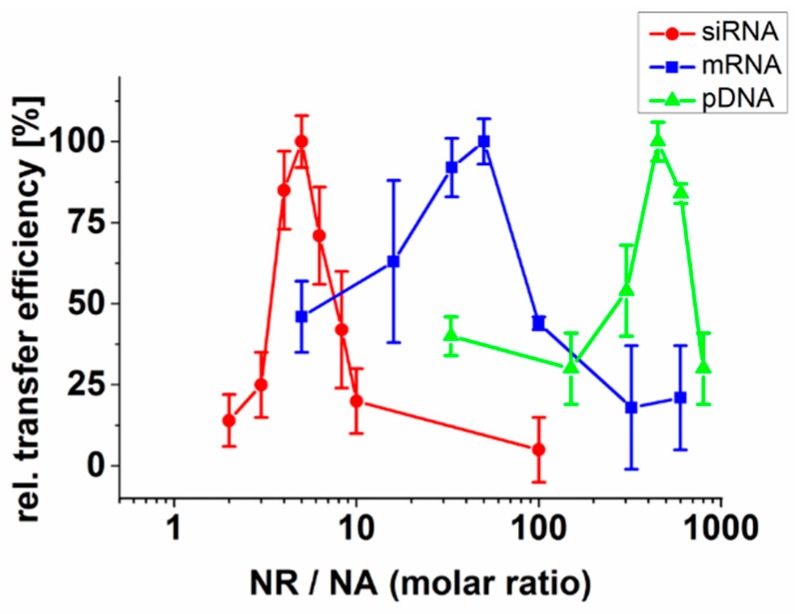
Influence of nucleic acid size on transfer efficiency. Nucleic acids (NA) with different sizes (anti-GFP siRNA, mRNA (GFP-mRNA), and GFP-expression plasmid (pDNA)) were complexed with the neutralization reagent (NR) protamine before incubation with FLs. Depending on the NR/NA molar ratio, NA transfer efficiencies were determined as the number of GFP positive cells after mRNA and pDNA transfer. For siRNA, the knockdown efficiency of eGFP–mRNA was determined by qRT–PCR. Maximum transfer efficiencies were set to 100% for best visibility. Values are given as mean with standard deviation of three independent measurements. NR = neutralization reagent; NA = nucleic acid.

Choosing protamine as NR, we analyzed the effect of pre-incubation on DNA (eGFP-expression plasmid, pDNA), mRNA (eGFP–mRNA), and siRNA (eGFP-silencer-siRNA) species. NR/NA molar ratios were varied from 2 to 1000. After incorporation of neutralized NAs and subsequent transfer into CHO cells, transfer efficiencies were determined by either measuring the reduction of GFP expression in the case of siRNA or by analyzing the number of GFP positive cells after pDNA and mRNA transfer, respectively (Figure 1). The data indicate that partial neutralization of NAs strongly enhances their transfer by membrane fusion. Interestingly, depending on the nucleic acid type, very different NR/NA ratios resulted in the best transfer.

When calculating the amount of nucleotide per NR, the optimum amount of NR correlated with NA size (see also Table 1) with 4–6 siRNA nucleotides per NR molecule, 20 mRNA nucleotides and 16 pDNA nucleotides per NR-molecule, respectively.

**Figure 2:**
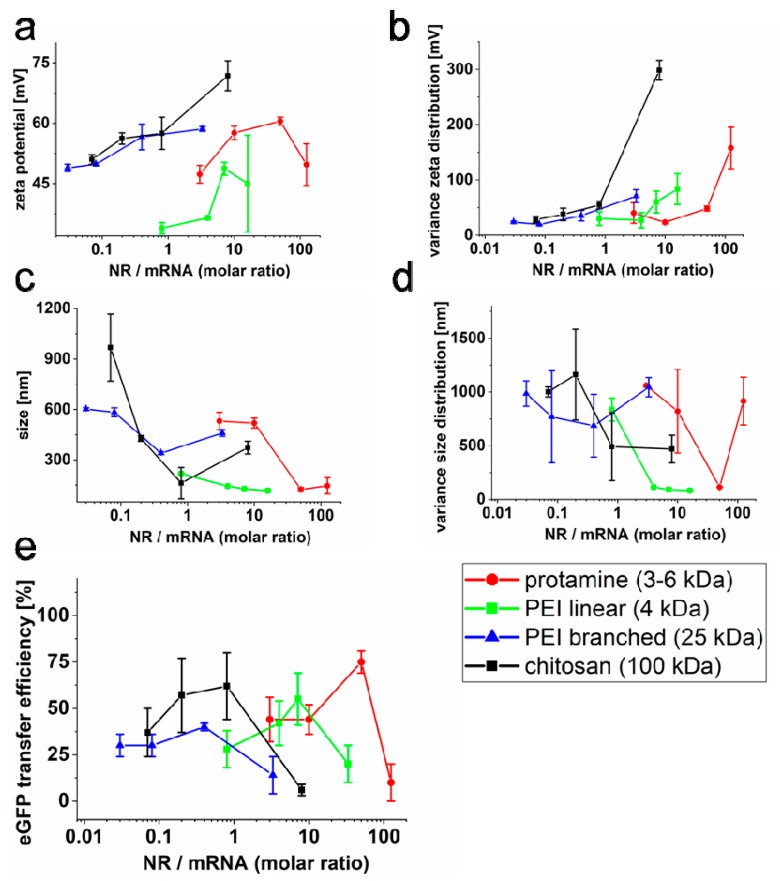
Influence of neutralization reagents and ratios on liposomal complex charge and size. Protamine, linear, and branched PEI and chitosan were tested as NR to efficiently incorporate NA into FLs and enable the efficient transfer of mRNA. Zeta potentials (**a**), variance of zeta potentials (**b**), complex size (**c**), and variance of complex sizes (**d**) are the most prominent characteristics that influence transfer efficiencies of FLs (**e**). Highest transfer efficiencies were achieved with maximal high zeta potentials at the lowest zeta potential variance in combination with the smallest complex sizes and size variances for all four NRs. NR = neutralization reagent; NA = nucleic acid.

We characterized the underlying mechanism in more detail by zeta potential measurements on cargo loaded FLs (DOPE/DOTAP/DiR 1/1/0.1) at various NR/NA ratios. For simplicity reasons and strong transfer sensitivity to suboptimal fusion conditions, we focused on GFP-mRNA as NA. Since various neutralization agents for NAs are described that differ in charge density and size, we compared four positively charged polymers with sizes ranging from 3 kDa (protamine), 5 kDa, and 25 kDa (unbranched and branched polyethylenimine (PEI)) to 100 kDa (chitosan). While FLs in the absence of mRNA exhibited a well-defined zeta potential in the range of 65 mV (Figure S1a), FLs incubated with mRNA without previous neutralization showed strongly reduced zeta potentials of around 40 mV. Low NR/mRNA molar ratios led to clearly reduced zeta potentials between 34 and 51 mV in complex with FLs. In contrast, high neutralization ratios resulted in very broad zeta potential distributions with several peaks or shoulders (Figure S1c). For all NRs, best mRNA transfer efficiencies were found for FL complexes with the highest zeta potential (Figure 2a), lowest zeta potential variance (Figure 2b), minimal complex size (Figure 2c), and smallest size variance (Figure 2d). Such complexes were most similar to free FLs. Furthermore, such optimal fusion conditions went along with strong but still incomplete neutralization of nucleic acids (−5.6 mV at mixing ratio of 50:1), as shown for protamine/mRNA complexes in the absence of FLs (Figure S1b).

Comparable results were found for all neutralization reagents tested. After incubation with eGFP–mRNA at NR/mRNA molar ratios from 0.03 to 100 and subsequent incubation with FLs, transfer efficiencies exhibited distinct optimum ratios again. Furthermore, the data argued for a decrease of the optimum NR/mRNA ratio with increasing size of the neutralization molecule (Figure 2b and Table 2).

**Figure 3:**
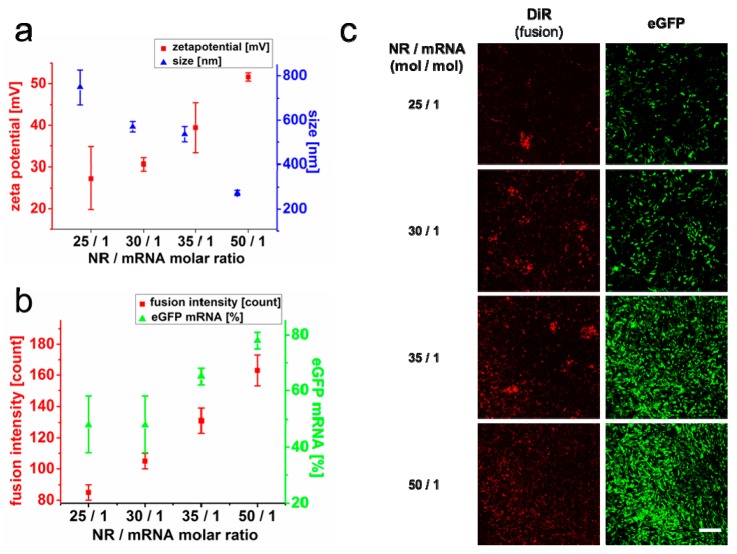
Effects of nucleic acid neutralization on liposomal complex characteristics and cellular transfer efficiency. Chinese hamster ovary cells (CHOs) were treated with FLs loaded with twofold eGFP–mRNA amounts previously neutralized with different amounts of protamine as NR to enhance the size effect of the protamine-neutralized mRNA complexes. Particle size and zeta potential were determined by dynamic and electrophoretic light scattering, respectively (**a**), and are well comparable to the corresponding ratios given in Figure 2. Fusion intensity as well as protein expressions (eGFP) were quantified by flow cytometry (**b**) and visualized by confocal microscopy recording the fluorescence emission of the membrane tracer DiR (red) and eGFP (green) (**c**). Values are given as mean with standard deviation of three independent measurements. Scale bar, 250 µm. NR = neutralization reagent.

For detailed neutralization analysis, twofold concentrations of mRNA were incubated with protamine at varying molar ratios (25/1 to 50/1) and subsequently added to identical FL-concentrations, as indicated above. Protamine was chosen due to its strongest sensitivity in respect of transfer efficiency from 10% to around 80% at different ratios (Figure 2e). For all mixtures, we characterized FL-sizes and zeta potentials as well as fusion intensities and eGFP–mRNA expression levels. The latter two increased monotonically with increasing concentrations of protamine. Interestingly, best NR/mRNA ratios for mRNA transfer correlated not only with high zeta potentials in the range of 52 mV as shown before but also with small FS/NR/mRNA complex sizes (275 nm; Figure 3a). Vice versa, reduced and therefore suboptimal protamine concentrations went along with decreasing zeta potentials, enlarged, precipitating fusogenic complexes that were already well visible by phase contrast microscopy (see Figure S2) and went along with lowered fusion and mRNA transfer efficiencies (Figure 3b,c).

Since NA neutralization by protamine had pronounced effects on liposome size and function, we explored these correlations by changing the pH-value of the NR buffer and, therefore, protamine charge (protamine isoelectric point around pH 12) without altering overall system composition and NR/mRNA ratio (constant at 50/1). Data show that protamine at lowered pH-values (pH 8 instead of pH 11) had similar effects as reduced NR/mRNA ratios with significantly lower zeta potentials, enhanced FS/NR/mRNA complex size, and impaired fusion and mRNA transfer efficiency (Table 3).

### 2.2. Effect of the Liposomal Formulation on Nucleic Acid Transfer Efficiency

**Figure 4:**
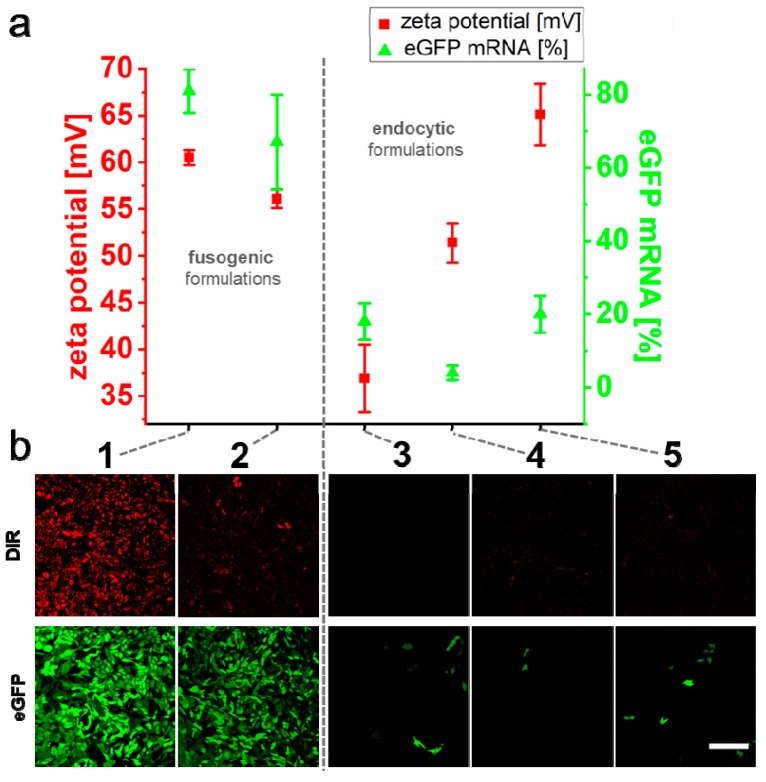
Influence of liposomal formulation on membrane fusion and nucleic acid transfer efficiencies. The liposomal formulations DOPE/DOTAP/DIR (mixing ratio 1/1/0.1) (1), DOPE/MVL-5/DIR (1/0.2/0.1 and 1/1/0.1) (2 and 5), DOPE/DOTAP (1/1) (3), and DOPC/DOTAP/DiR (1/1/0.1) (4) were used to deliver eGFP–mRNA into CHO cells. Protamine was used as a neutralization reagent. The average zeta potential of liposomes in complex with partially neutralized nucleic acid (see also Table S1) and subsequent eGFP–mRNA transfer efficiency are shown as mean with s.d. of three independent measurements (**a**). Fluorescence micrographs of CHO cells 24 h after treatment with the same complexes were recorded to monitor membrane fusion (DiR) and eGFP transfer efficiencies (eGFP), respectively (**b**). Scale bar 200 µm.

As demonstrated before, fusion efficiency is strongly dependent on the chemical nature and mixing ratio of FL–lipid components [43]. To elucidate the impact of liposomal composition on nucleic acid transfer, we first tested if removal of the fusion inducing lipid analog DiR would also influence nucleic acid transfer after complexation with protamine (NR/NA 50/1). All investigations were continued with mRNA as NA due to its highest transfer sensitivity to suboptimal fusion conditions. Results show that liposomal formulations with DOPE and DOTAP (1/1) were barely able to transfer eGFP–mRNA into cells with efficiencies below 20% as compared to 80% in the presence of DiR based on flow cytometry measurements (Figure 4a,b).

In the next step, we replaced DOTAP in our optimal standard formulation (DOPE/DOTAP/DiR, 1/1/0.1) by MVL-5. This multivalent cationic lipid contains five positive charges on its head group instead of only one for DOTAP. For optimal comparison, we kept once the lipid molar ratio (1/1/0.1) and once the calculated net surface charge (1/0.2/0.1) constant. With similar surface charge densities of MVL-5 liposomes (1/0.2/0.1), mRNA transfer was well possible with efficiencies in the 70% range. Although efficiencies were high, fusion intensities were reduced, and eGFP transfer efficiencies showed higher variabilities compared to DOPE/DOTAP/DIR preparations. These transfer and fusion efficiencies correlated with lowered zeta potentials in the range of 56 mV (Figure 4a). Interestingly, elevated MVL-5 concentrations (1/1/0.1) inhibited liposomal fusogeneity. This effect went along with low mRNA transfer, although zeta potentials were even slightly higher than those of standard, DOTAP-containing FLs (Figure 4a,b). 

Additionally, the neutral lipid DOPE of FLs was exchanged by DOPC. This exchange was described to abolish liposomal fusogeneity [43] and reduced the zeta potential of liposomes after incubation with NR/NA complexes to 51 mV. Furthermore, fusion events after contact with CHO cells were not detectable, and eGFP expression levels were down to just 4% (Figure 4a,b). Live-cell microscopy analyses indicated that in the presence of NR/NA complexes, only DOPE/DOTAP/DiR (1/1/0.1) and DOPE/MVL-5/DiR (1/0.2/0.1) liposomes were fusogenic, while all other formulations were mainly taken up by endocytosis (supplementary movie M1).

### 2.3. Quantification of mRNA Transfer on Single Cell Level

**Figure 5:**
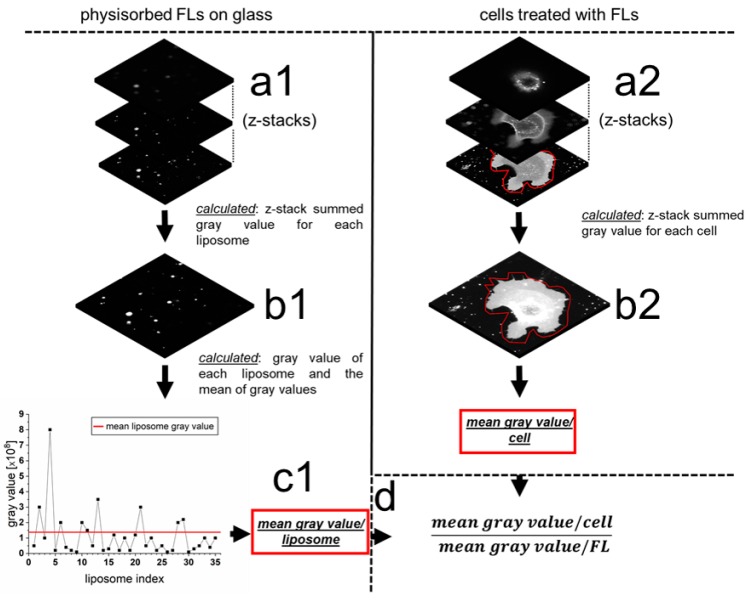
Schematic overview of the membrane fusion events quantification. The fluorescence signal of mRNA loaded FLs adsorbed on glass was recorded in 3D in the red channel (DiR) (**a1**). Gray values of the z-stacks were summed up for each individual liposome (**b1**). In addition, a mean gray value of all detected liposomes was calculated (**c1**). Subsequently, nHEK cells, previously treated with protamine/eGFP–mRNA/FL complexes, were imaged in 3D (**a2**), and gray values of the z-stacks were summed up for each single cell (**b2**). Liposomal gray values were analyzed in 12 independent measurements with 900 liposomes in total and compared with mean gray values of 15 cells. Using the values calculated in (**c1)** and (**b2)**, the total number of liposomes fused with a single nHEK cell was calculated (**d**). FL = fusogenic liposome.

To further characterize fusion-dependent nucleic acid transfer, we estimated the effective number of NR/NA-loaded liposomes that fused with nHEK cells. Based on average gray values of physisorbed DOPE/DOTAP/DiR (1/1/0.1) fusogenic liposomes that were complexed with protamine as NR and eGFP–mRNA as NA (50/1 ratio) and summed gray values per nHEK cell after fusion with such liposomes, we calculated 1700 (s.d. 700) fusion events per cell within 10 min (Figure 5). 

In addition, the mRNA amount transferred into the cell cytoplasm was determined by qRT–PCR to be 10^5^ mRNA molecules per cell after 10 min of fusion. Our results showed that 0.5% of the total mRNA quantity was transferred into cells via fusion. As control formulation, the classical transfection reagent Lipofectamine MessengerMax was used to transfer the same mRNA into nHEK cells by endocytosis. Here we found that only half the amount (0.25% of the total mRNA) was successfully internalized into nHEK cells, although incubation time was already prolonged to 30 min compared to 10 min for fusion.

### 2.4. mRNA Delivery Into Primary Cortical Neurons and Freshly Isolated Neural Tissue

Most NA transfer reagents exhibit reduced efficiencies when used on primary cells [44]. Efficient transfer into primary tissue is even more challenging, and viral transduction was described as likely the only method that results in sufficient transfer of nucleic acids [45]. To test if similar effects would also occur for fusion dependent NA transfer, DOPE/DOTAP/DiR (1/1/0.1) fusogenic liposomes were used to transfer protamine neutralized eGFP–mRNA (50/1 ratio) into freshly isolated cortical neurons from rat embryos. mRNA was chosen due to its tremendous medical potential and fast readout ability that allow short incubation times of isolated tissues before analysis.

**Figure 6:**
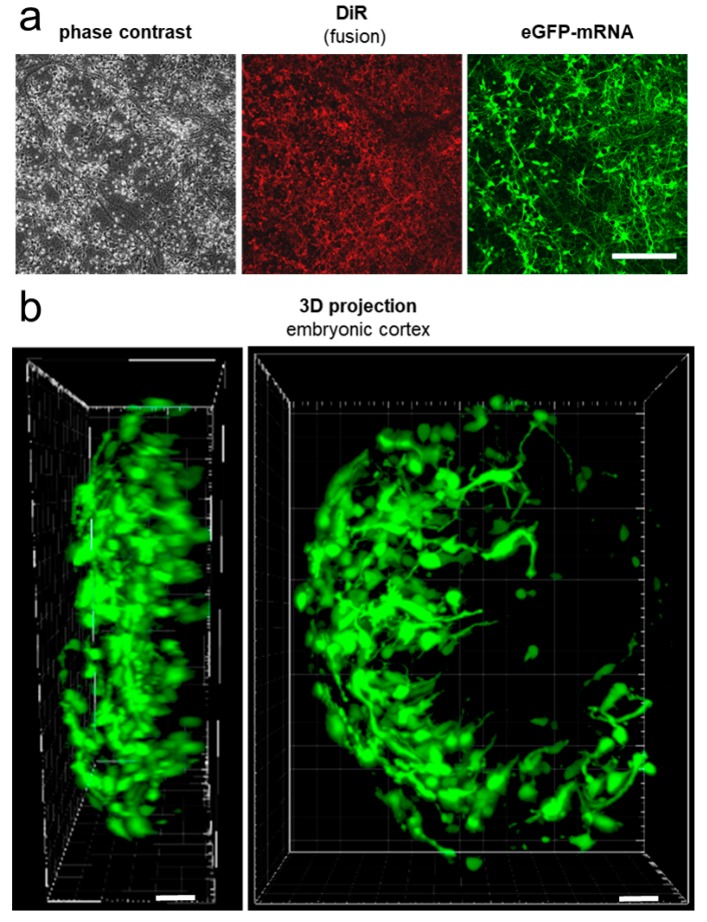
eGFP–mRNA transfer into primary neuronal cells and primary cortical tissue using FLs. Fluorescence and phase contrast micrographs of primary rat cortical neurons six days after treatment with protamine/eGFP–mRNA/FLs (**a**). 3D reconstruction of rat embryonic cortex based on eGFP fluorescence. eGFP–mRNA was delivered by FLs 6 days before analysis (**b**). Scale bars 200 µm.

As shown in Figure 6a, FL/NR/NA complexes were highly fusogenic also on these primary cells. Furthermore, fusion resulted in the efficient transfer of eGFP–mRNA and GFP signals that were detectable for more than 1 week after treatment. Fusion visibly changed neither cell morphology nor cell viability and allowed normal neurite outgrowth and neuronal network formation. 

Additionally, we treated freshly isolated rat embryonic cortical tissue with the same FL/NR/NA complexes and observed comparably high eGFP–mRNA transfer efficiencies 24 h after fusion (Figure 6b). As in the case of cell lines, cell morphology was visibly unaltered. 

## 3. Discussion

Transfer of NAs into mammalian cells is a well-established technique in medical and pharmaceutical research. However, spontaneous incorporation of NAs is hampered by the repulsive electrostatic forces acting between the negatively charged NA molecules and the also negatively charged glycocalyx on the cell surface. Therefore, NA neutralization strategies have been applied frequently to enhance NA internalization efficiency [46,47,48]. For this purpose, nucleic acids are typically complexed by positively charged peptides [16,49,50,51], synthetic polymers [18,19,52,53], or cationic liposomes [41,46,54] as neutralization reagent. NR and NA interaction switches the net complex charge from negative to positive, enabling an attractive interaction with the cell surface, and, consequently, their cellular uptake [52].

Our experiments showed complexation of NA with FLs in the absence of NR, albeit, NA transfer efficiency remained very low. We hypothesized that the strong electrostatic interactions between NA and FL blocked efficient interaction between FLs and cell surfaces and therefore impaired NA transfer into the cytoplasm. To overcome this barrier, we characterized a new delivery strategy by pre-incubation of NA with another positively charged molecule before incorporating them into FLs. Applying this method, NA/NR complexes served as cargo and FLs as delivery vehicles. As already clearly shown for FLs in the absence of NR molecules and NAs [34,55], parallel experiments on NA/NR/FL complexes also proved fusion-based transfer and high biocompatibility [38]. Underlying liposomes (there named Fuse-It-mRNA) had the identical composition as characterized here as optimum (DOPE/DOTAP/DiR 1/1/0.1) and were unaffected in effectiveness by various endosomal uptake blockers while keeping natural cell functions of various cell types completely unaffected.

For optimal results, a precisely adjusted charge balance between the components NA, NR, and FLs plays a crucial role in homogenous complex formation and subsequent efficient cargo delivery (Table 1). Furthermore, large neutralization reagents like chitosan or branched PEI are able to reduce the negative charge of nucleic acids more effectively than small peptides like protamine, or linear PEI; therefore, less reagent is needed for the best complex formation (Table 2). Interestingly, other transfer methods that apply cationic polymers for NA neutralization usually need the polymer in high excess [8,21] to completely compensate or even revert the negative charge of NA. Rettig et al. exemplarily demonstrated that molar ratios of NR (protamine)/mRNA of around 400/1 resulted in best endosomal-dependent NA transfer efficiencies [56]. While similar ratios were found here for fusion-driven pDNA transfer, lower ratios were identified for mRNA (50/1) and siRNA (5/1) that furthermore resulted in just partial neutralization (Table 1). Although reduced ratios for RNA species can be explained by smaller molecule sizes and higher flexibilities to some extent [57,58], also the different type of incorporation mechanisms most likely plays an important role. This is because endosomal uptake is barely affected by molecule charge, and efficient neutralization of NA and high excess of NR is necessary to allow for cell surface interaction [8,18,21,59]. In contrast, for fusion driven NA transfer, NR/NA interaction with FL as well as FL/NR/NA contact to the plasma membrane are largely dependent on electrostatic interactions, as also shown for FL dependent protein transfer [35]. Our results indicate (Figure 2 and Figure 3) that high excess of protamine impairs interaction of NR/NA to FL and absence or just low amounts of protamine inhibit FL fusogeneity. In between these opposing conditions for RNA delivery, a maximum occurs. At this optimum, NAs are still just partially neutralized to further enable the interaction with FLs (see Figure S1b), and the resulting cargo loaded FLs show zeta potentials of 40–45 mV whereas bare FLs typically exhibit values in the range of 60–65 mV.

As described in literature, FL composition strongly influences fusogeneity [43]. In line with this, we identified FLs formed from the lipid mixture of (DOPE/DOTAP/DiR 1/1/0.1) as an optimum vehicle for RNA transfer. Moreover, in agreement with literature [34,43], liposomes lacking the aromatic lipid analog DiR or containing a phosphocholine lipid instead of phosphoethanolamine were non-fusogenic. This loss of function went along with low NA transfer efficiencies, indicating that the alternative endosomal uptake routes are of negligible efficiency. This was also proven by unaffected transfer efficiencies for the optimized system identified here upon the use of different endocytosis blockers [38]. Notably, when the cationic lipid DOTAP was replaced at a 1/1 ratio by MVL-5, liposomal delivery capacity drastically decreased as well, although FL zeta potential remained highly positive with values close to 55 mV.

As proposed by Kolasinac et al., not only the charge but also the shape of lipid molecules forming FLs might play a crucial role in fusion [43]. Here, the excess of molecules with inverted conical (MVL-5) or cylindrical shape (DOPC) seems to interfere with phase behavior and drastically reduced liposomal fusion ability. Reduction of MVL-5 concentration in the membrane by 80% (zeta potential 51 mV) reduced the total number of positive surface charges to values found for classical DOTAP containing FL (zeta potential of 58 mV) and, intriguingly, resulted again in high mRNA transfer efficiency (70%). Interestingly, fusion efficiency largely depends on high concentrations of DOTAP. While concentrations of 25% and lower massively impair fusogeneity, concentrations above 50% continuously result in high fusion efficiencies of 75% and more. Furthermore, cell culture experiments indicate that the transfer of such high concentrations of positively charged lipids by fusion does not induce cytotoxic effects [33] as typically found for other systems [60]. As described by Chernomordik and Kozlov, membrane fusion intermediate states require membrane leaflets with slightly negative curvatures. Lipids like DOTAP or DOPE with conical effective shapes tend to form such surfaces, while molecules with cylindrical or inverted conical shapes build membranes with positive curvatures [61]. In these cases, hemi-fusion pore formation, as a fusion intermediate state, is not favored energetically, and, therefore, fusion processes might be inhibited. This scenario is seemingly questioned by our results on the cationic lipid MLV-5. Even though it exhibits conical shape, FLs formed from a lipid mixture containing 15% (mol/mol) MVL-5 exhibit very high fusion efficiency. Presumably, at such low concentrations, the molecular shape of the majority molecule DOPE (inverted conical) dominates phase behavior.

All effects described above allow efficient interaction of NR/NA treated FL with the cellular plasma membrane and, as a consequence, the fusion of more than 1500 liposomal complexes per cell within 10 min. Such numbers are significantly higher than those described for endocytosis-dependent processes with 1400 complexes interacting with the plasma membranes within 30 min and uptake of 850 complexes within this time [21]. However, fusion events per time calculated here are subject to a large uncertainty. This is because we cannot exclude fluorescent quenching effects [62,63] due to strongly differing fluorophore concentrations in FLs and plasma membranes after fusion. Additionally, putative differences in FL size distribution or complexation status that preferably bind to planar surfaces or are able to fuse with cellular membranes have been neglected. Nevertheless, while endosomal uptake rates seem to vary tremendously between different cells [21], data presented earlier indicate that due to mainly physicochemically driven underlying processes, fusion efficiency is largely independent on cell type [38].

Most importantly, high fusion rates went along with efficient NA transfer. These results indicate that most FL incorporated NR/NA complexes without losing their fusogenic potential. In the end, with approximately 10^5^ molecules, 0.5% of the applied mRNA amount have been transferred by fusion within 10 min. This amount is twice as high as what we and other groups could achieve with classical endosomal based transfection reagents at best conditions. Batard et al., for example, detected an NA uptake of 10^5^ molecules within 8 h using cationic endocytic liposomes, while 30 times more NA molecules were internalized within half the time using the same liposomes in the presence of calcium phosphate salt [64].

A remarkable result of this efficient, fusion-based NA incorporation directly into the cytoplasm is an unusually fast appearance of detectable protein amounts. For GFP–mRNA, first protein signals were already well detectable 1 h after fusion (see also [38]), while for classical lipid- or polymer-based transfection reagents, this takes several hours [65]. This makes fusion a versatile alternative to classical transfection reagents with high functionality in cell culture as well as tissue samples using identical fusion parameters. Further experiments will show if similar results can be achieved upon in vivo use.

## 4. Materials and Methods

### 4.1. Cell Culture

Chinese hamster ovary cells (CHO-K1) (ATTC, Manassas, VA, USA) and normal human epidermal keratinocytes (nHEKs) (Cell Systems, Troisdorf, Germany) were used to analyze nucleic acid delivery efficiencies by FLs. Before treatment, CHO-K1 cells were maintained in DMEM-F12 culture medium (Sigma-Aldrich, Darmstadt, Germany) supplemented with 10% fetal bovine serum and a 1/100 dilution of an antibiotic solution (10,000 units penicillin and 10 mg/mL streptomycin in 0.9% NaCl, (Sigma-Aldrich Darmstadt, Germany)). nHEK cells were cultured in DermaLife® K Keratinocyte Culture medium (CellSystems, Troisdorf, Germany) with manufacturer’s supplements lacking tumor necrosis factor (TNF). For experiments on cortical neurons, cells were isolated from rat embryos and cultured as described previously [66]. All cells were continuously kept at 37 °C and 5% CO2 in a humidified atmosphere. Cell confluence was kept below 80%. Then, 24 h before fusion 60,000 cells were seeded on fibronectin (BD Biosciences, San José, CA, USA) coated Petri dishes (Ø = 3.5 cm) (20 μg/mL fibronectin (BD bioscience) for 30 min at 37 °C) and cultivated.

### 4.2. Preparation of Liposomes

The neutral lipids 1,2-dioleoyl-sn-glycero-3-phosphoethanolamine (DOPE), and 1,2-dioleoyl-sn-glycero-3-phosphocholine (DOPC), and the cationic lipids 1,2-dioleoyl-3-trimethylammonium-propane (DOTAP), and N1-[2-((1S)-1-[(3-aminopropyl)amino]-4-[di(3-amino-propyl)amino]butylcarboxamido)ethyl]-3,4-di[oleyloxy]-benzamide (MVL-5) were purchased from Avanti Polar Lipids, Inc. (Alabaster, AL, USA). The fluorescent lipid analog DiR (1,1’-dioctadecyl-3,3,3’,3’-tetramethylindotricarbocyanine iodide) was ordered from Thermo Scientific (Waltham, MA, USA). The lipid components were homogeneously mixed with the lipid analog in chloroform at a molar ratio of DOPE (DOPC) / DOTAP (MVL-5) / DiR of 1/1/0.1 (mol/mol). Where indicated a molar ratio of 1/0.2/0.1 was used. For control samples, only the neutral and the cationic lipids without DiR were used at the same ratios. After component mixing, chloroform was evaporated at reduced pressure for 0.5 h. Subsequently, dried lipid films were dispersed in 20 mM 2-(4-(2-hydroxyethyl)-1-piperazinyl)-ethansulfonic acid (HEPES) buffer (pH 7.4) to a final lipid concentration of 2 mg/mL. These suspensions were vortexed for approximately 1–2 min and additionally homogenized in a standard ultrasonic bath for 10–20 min at 4 °C. Liposome stock solutions were stored at 4 °C until usage.

### 4.3. Preparation of Neutralization Reagents (NR)

Protamine (grade IV, Sigma Aldrich, Darmstadt, Germany), linear (product number 764582, Sigma Aldrich) and branched (product number 408727, Sigma Aldrich) polyethyleneimine (PEI) (Sigma Aldrich), and chitosan (100 kDa, Sigma Aldrich) were used as neutralization and complexation reagents for nucleic acids. Neutralization reagents (NRs) were dissolved in ultrapure water or in 0.2 M sodium acetate buffer pH 4.5 (chitosan) at a concentration of 1 mg/mL. In one experimental setup, the effect of reduced pH-values of the protamine containing NR reagent was analyzed by acidifying the solution with CO_2_ for 10 min at RT to result in a decreased pH-value from naturally pH 11 to pH 8.

### 4.4. Preparation of Nucleic Acid Containing Fusogenic Liposomes

Before experiments, liposomes were vortexed and homogenized by ultrasonication for 10 min at 4 °C. In parallel, 1 µg mRNA (eGFP mRNA, TriLink USA) or alternatively 30 pmol siRNA (eGFP silencer siRNA; Thermo Scientific) or 2 µg plasmid DNA (eGFP plasmid; BD Bioscience) were incubated with indicated amounts of NR (protamine, linear or branched PEI, and chitosan) for 10 min at RT in a total volume of 3–6 µL. Subsequently, neutralized nucleic acids were mixed with 2.5 µL of FLs and homogenized in an ultra-sonication bath for 5 min below RT in a total volume of 5.5–8.5 µL. Before cell treatment, solutions were diluted with 250 µL of PBS (137 mM NaCl, 6.2 mM Na_2_HPO_3_, 1.5 mM KH_2_PO_4_, pH 7.4) and sonicated for 2 min at 4 °C. Cells were incubated with NA/NR/FLs complexes for 5–15 min at 37 °C. After treatment, the liposomal solution was replaced by cell culture medium.

### 4.5. Quantification of Knock Down Efficiency of Transiently Expressed eGFP

For quantification of siRNA transfer efficiencies, CHOK1 cells were transfected with 1 µg of eGFP plasmid using Lipofectamine2000 reagent one h before siRNA transfer via FLs. Fusion was performed for 10 min as described above, using 30 pmol Ambion eGFP silencer siRNA (Thermo Fisher Scientific, Waltham, MA, USA). eGFP protein levels were quantified by flow cytometry (see below) 24 h after siRNA treatment.

### 4.6. mRNA Transfer Into Isolated Cortical Brain Tissue

Cortical tissue was isolated from 19-day-old Wistar rat embryos by dissection as described before [66] and subsequently fused with the following complex: 2 µg eGFP–mRNA, 1 µg protamine, 2.5 µL FLs, and 250 µL PBS for 30 min at 37 °C. Complex preparation was performed as described above. After fusion, the solution was replaced by prewarmed neurobasal media (Thermo Fisher Scientific, Waltham, MA, USA), supplemented with GlutaMAX (Thermo Fisher Scientific, Waltham, MA, USA), B-27 (Thermo Fisher Scientific, Waltham, MA, USA), and gentamicin (Sigma, Taufkirchen, Germany) and tissue fragments were cultured for 4–6 days at 37 °C and 5% CO_2_ containing, humidified atmosphere. The experiments were approved by the local ethics committee (animal testing permission No. 84-02.04.2015.A173 (Landesamt für Natur, Umwelt und Verbraucherschutz NRW)). For 3D image reconstruction of eGFP-expressing cortical tissue, the Imaris-software (Bitplane, Zurich, Switzerland) was used. 

### 4.7. Zeta Potential and Size Distribution Measurements

Particle size and zeta potential were determined by dynamic and electrophoretic light scattering, respectively, using a zetasizer (Nano ZS from Malvern Instruments, Malvern, U.K.) equipped with a HeNe laser (633 nm). Scattered laser light was collected at a constant angle of 173°. Prior to measurements, NA/NR/FLs complexes were diluted 1/10 for size and 1/100 for zeta potential measurements with degassed ultrapure water. All measurements were performed at 20 °C and repeated three times at 1 min intervals. Data were collected from three independent samples. Data were analyzed by the manufacturer’s software. Zeta potential measurements were performed with a refractive index of 1.33. Size was determined by intensity.

### 4.8. Light Microscopy

Live cell analyses were performed at 37 °C and 5% CO_2_ using an inverse confocal laser scanning microscope (cLSM 710, Carl Zeiss Micro-Imaging GmbH, Jena, Germany) equipped with an argon ion laser (488 nm) and a helium-neon laser (633 nm). eGFP and DiR were detected using appropriate filter settings. Depending on experimental needs, one of the following objectives (all Carl Zeiss) was used: EC Plan-Apochromat 20×/0.8 Ph2, EC Plan-Neofluar 63×/1.25 Oil Ph3, or EC Plan-Neofluar 10×/0.30 Ph1. Overview images were recorded in all cases at the center of the substrates.

### 4.9. Flow Cytometry

NA-transfer efficiencies were characterized by flow cytometry (GUAVA 8HT, Merck Millipore, Billerica, MA, USA). Prior to analysis, cells were trypsinized (Trypsin–EDTA solution from Sigma-Aldrich, Darmstadt, Germany) 24 h after NA delivery, resuspended in 200 µL of cell culture medium and analyzed without fixation. For each sample, at least 10,000 cells were analyzed for cell morphology (granularity and size), fusion efficiency (DiR), and eGFP-expression. Suitable gates were chosen in the forward scatter vs. side scatter dot plot. The fluorescence signal of the lipid tracer DiR was excited with the 640 nm laser line and collected in the NIR2 channel using a band pass filter BP 785/70 nm. eGFP signal was excited with the 488 nm laser line and recorded in the green channel through the band pass filter BP 525/30 nm. Fusion intensities were determined as mean fluorescence intensity over all DiR positive cells. Fusion efficiencies represent the percentage of DiR labeled cells of all cells. Transfer efficiencies for mRNA and plasmid DNA (both encoding eGFP) were determined as the ratio of eGFP positive cells of all analyzed cells. Flow cytometry thresholds were set based on controls and kept stable. siRNA knockdown efficiencies were quantified as the number of eGFP positive cells after transient eGFP plasmid transfer in eGFP silencer siRNA treated cells compared to cells without siRNA treatment.

### 4.10. Quantitative Analysis of Membrane Fusion Events

The number of fused liposomes per cell was determined from confocal images. To this end, the average fluorescence intensity (defined by detected and summed gray values) of fusogenic liposomes and the averaged total fluorescence intensity per cell were analyzed after treatment with FLs.

To determine the average liposomal fluorescence intensity, 2.5 µL of FL solution was diluted 1:400 in PBS, and 250 µL of the diluted solution was placed on a cover slide for 2 min. This resulted in well-separated, physisorbed liposomes on the slide. For each liposome, the overall fluorescence intensity was recorded by confocal z-stack imaging (cLSM 710, Carl Zeiss). A manually defined gray value was used as a threshold to segment the liposomes. Average intensities were determined in nine independent experiments with at least 35 liposomes each, and the ensemble average was determined.

To quantify liposomal fusion events per cell, freshly FL treated nHEK cells were imaged in 3D to visualize the liposomal marker dye DiR. Cell borders were determined and used as masks. Gray values within the mask were summed up and are defined as fusion intensities. The total number of liposomes fused per cell was subsequently calculated as the ratio of overall fluorescence intensity per cell and the average liposomal fluorescence intensity. An analysis was performed on more than 15 independent cells. Throughout all experiments, microscope setups, as well as substrate conditions, were kept identical. As internal, reliable control, unfused liposomes next to the cells were used and showed comparable intensities as identified before for liposomes alone.

### 4.11. RNA Isolation and cDNA Synthesis

For RNA quantification, an RNeasy Plus Mini kit (QIAGEN GmbH, Hilden, Germany) was used according to the manufacturer’s recommendations. Isolation was performed 0.5, 6, and 24 h after mRNA transfer for quantification of transferred eGFP–mRNA and 24 h for quantification of mRNA knockdown in case of siRNA transfers. RNA yields were determined by measuring the absorbance of the RNA solution at 260 nm (A260) using an UV/VIS spectrometer (Nanodrop products, Wilmington, NC, USA). cDNA synthesis was performed using the QuantiTect Reverse Transcription Kit (QIAGEN GmbH, Hilden, Germany). For the quantification of eGFP–mRNA amounts that were transferred by fusion, 10 ng eGFP–mRNA was transcribed into cDNA as control for subsequent qRT–PCR experiments. All substrates were washed three times with PBS before RNA isolation to remove not internalized liposome–mRNA complexes from the cell surface. 

### 4.12. RT-PCR Assays

Synthesized cDNA was diluted 1/5 in RNAse free water before RT–PCR analysis. Subsequent qRT–PCR experiments were performed using an eGFP TaqMan® Assay (Thermo Scientific, Waltham, MA, USA) and a TaqMan® master mix. Glyceraldehyde 3-phosphate dehydrogenase (GAPDH) served as internal standard. Analysis was performed with a StepOne™ Real-Time PCR System (Thermo Scientific, Waltham, MA, USA) and evaluated by StepOne™ software (version 2.0.2). 

### 4.13. Statistical Analysis

Data are given as mean (s.d.). Analysis of variance (ANOVA) was used for multiple comparisons. A *p*-value of ≤0.05 was considered significant.

## 5. Conclusions

Fusogenic liposomes are highly efficient carriers for transferring any kind of nucleic acid into mammalian cells. Based on their high fusion ability with the cellular plasma membrane, they deliver nucleic acids directly into the cell cytoplasm. Due to the fast and efficient delivery process and low cargo degradation, protein expression can first be observed already 1 h after treatment. High biocompatibility and low toxicity, as shown in parallel experiments [38], make the optimized FL system developed here a promising candidate for future biomedical and pharmacological applications for the transfer of complexed nucleic acids.

## Figures and Tables

**Table 1:** Neutralization of nucleic acids (NA) with protamine as neutralization reagent (NR). Shown are the analyzed NAs, their sizes in nucleotides, the most efficient molar ratios of NR/NA as well as the corresponding numbers of nucleotides per NR.

NA	NA Length (Nucleotides)	Molar Ratio of NR/NA	Nucleotides/NR
siRNA	20–30	5/1	4–6/1
mRNA	1000	50/1	20/1
pDNA	4700	450/1	16/1

**Table 2:** Neutralization of mRNA with neutralization reagents (NR). The analyzed NRs, their sizes, and the most efficient molar ratios of NR/mRNA (NA) are given.

NR	Size [kDa]	NR/NA [mol/mol]
PEI branched	25	0.4/1
chitosan	100	0.8/1
PEI linear	10	10/1
protamine	3–6	50/1

**Table 3:** Effect of pH on NR/NA/FL (neutralization reagent/nucleic acid/fusogenic liposome)-complex characteristics. Shown are the zeta potentials, the sizes of the protamine/eGFP–mRNA/FL complexes at two different pH values, as well as the resulting fusion and transfer efficiencies after treatment of Chinese hamster ovary (CHO) cells. Values are given as mean with standard deviation (s.d.) of six (zeta potential), respectively, five (size) measurements. All values for pH 8 and pH 11 are significantly different.

pH of NR Buffer	Zeta Potential ± s.d. (mV)	Size ± s.d. (nm)	eGFP Transfer Efficiency ± s.d. (%)	eGFP Fluorescence Intensity ± s.d. (counts)
11	60.5 ± 0.3	141.5 ± 36	54 ± 7	475 ± 117
8	54.5 ± 3	344.8± 20	46 ± 3	230 ± 88

## Supplementary Materials

Supplementary materials can be found at https://www.mdpi.com/1422-0067/21/6/2244/s1.
