# Supplementary material for: Complex Size and Surface Charge Determine Nucleic Acid Transfer by Fusogenic Liposomes
Source: Int J Mol Sci. 2020 Mar 24;21(6):2244. doi: 10.3390/ijms21062244 (PMC7139958; doi:10.3390/ijms21062244)
Supplement: Supplementary file 1 [file ijms-21-02244-s001.zip › ijms-707684 supplementary-final/Supplementary Material.docx]

**Supplementary Material**


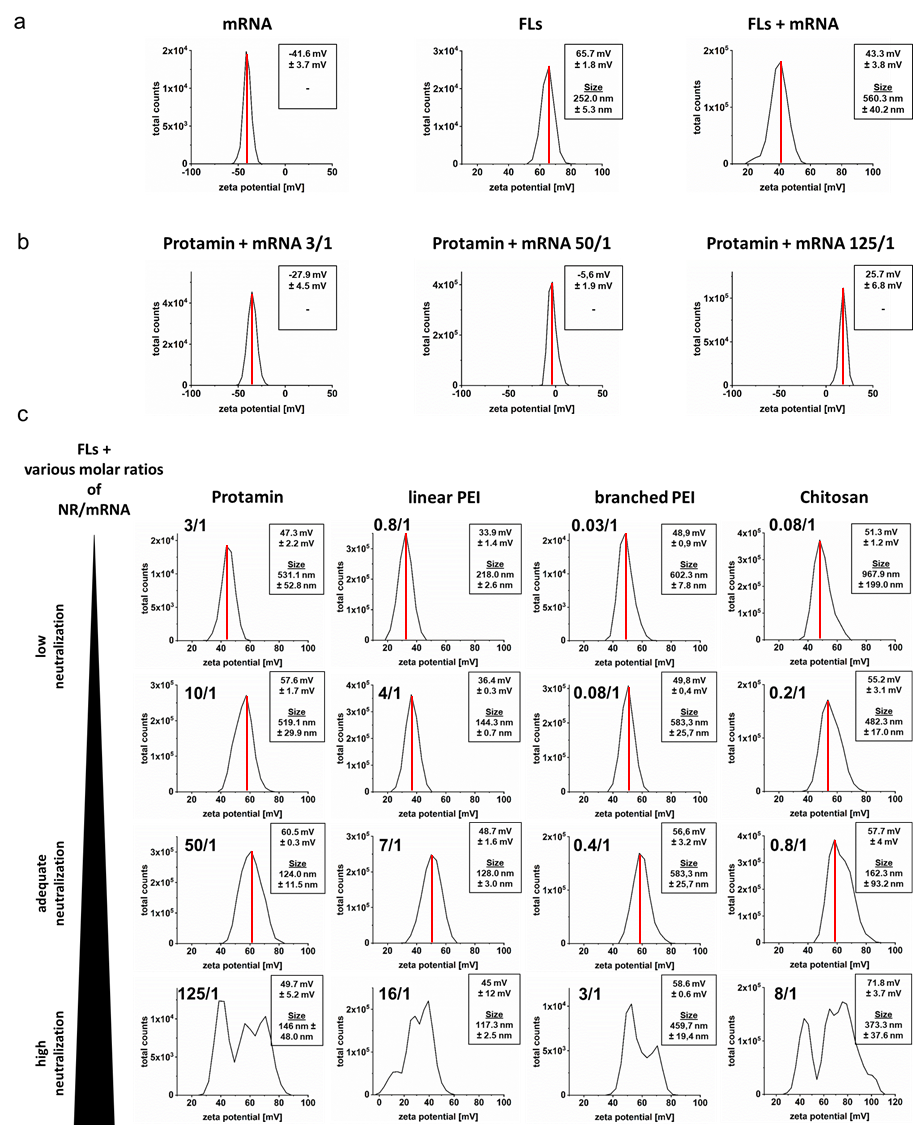


**Supplementary Figure S1**. Physicochemical characterization of fusion-involved compounds. Zeta potential measurements and size characterizations wherever possible, were performed for mRNA, fusogenic liposomes (FL) and mixtures of both (a). Zeta potential measurements were performed for RNA complexes with varying concentrations of protamine as neutralizing reagent (b). In (c) influence of neutralization reagents with increasing concentration on liposomal complex homogeneity and zeta potentials were characterized. Four NRs, i.e. protamine, linear and branched PEIs, and chitosan with different molecular weights were tested at varying concentrations (NR/mRNA ratios, indicated as numbers in each graph) to partially neutralize eGFP-mRNA and to complex it with FLs. Resulting zeta potential distributions are plotted. Red lines indicate distribution maxima in case only one maximum is present. Highest zeta potentials with lowest peak distributions and smallest complex sizes resulted in highest transfer efficiencies (adequate neutralization in third line for each NR).


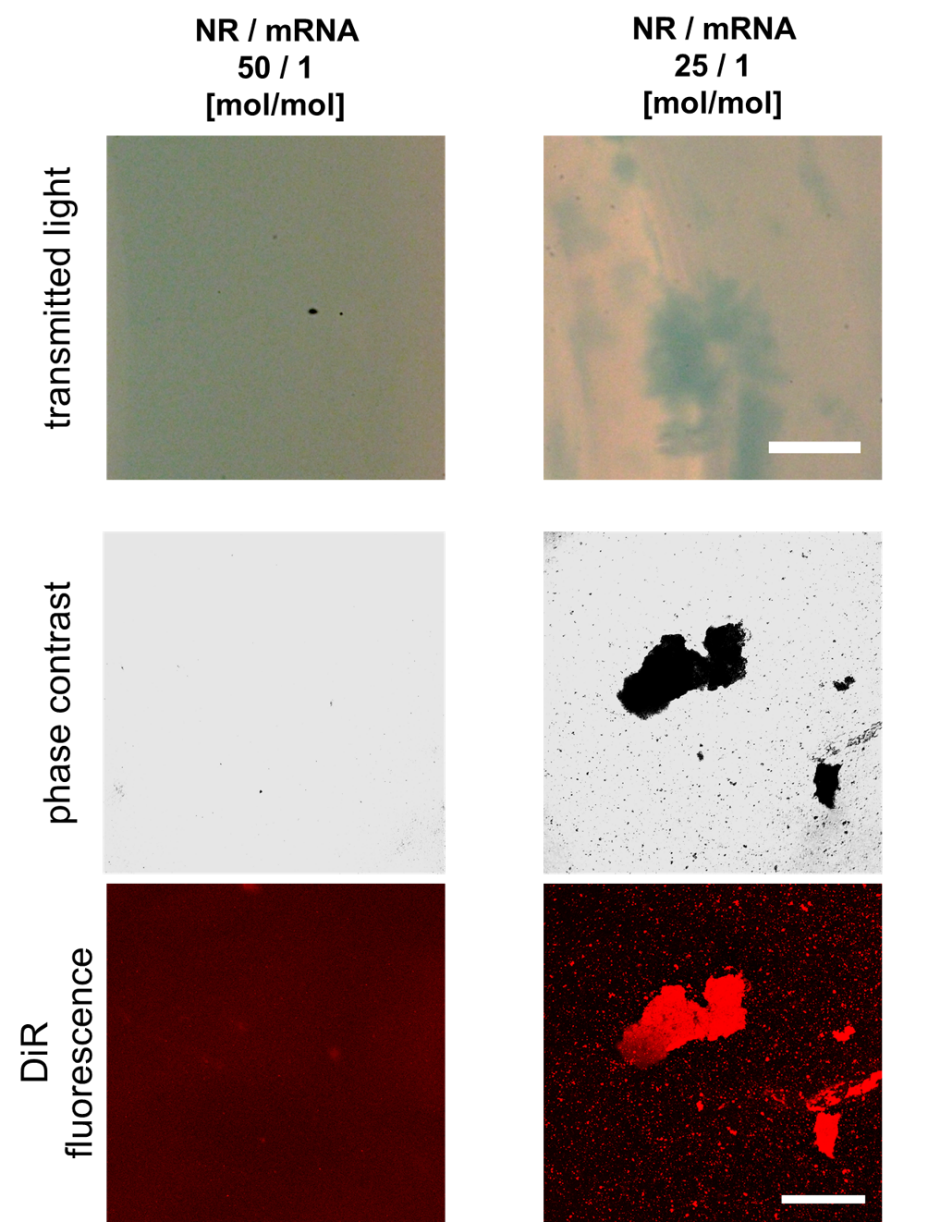


**Supplementary Figure S2**. Complex homogeneity of mRNA fusogenic liposomes depends on the NR/NA molar ratio. Transmission, phase contrast and fluorescence micrographs of protamine/eGFP-mRNA/FLs complexes after sedimentation on a glass surface. Protamine/mRNA molar ratios of 50/1 and 25/1 are shown. NR/mRNA ratio of 50/1 mol/mol resulted in homogeneous complexes without any aggregates while decreasing NR amount (protamine/mRNA=25/1) yielded inhomogenous complexes with large aggregates. Scale bars, 200 µm.

**Supplementary** **Movie M1:** Influence of liposomal formulations on membrane fusion. The liposomal formulations DOPE/DOTAP/DIR (mixing ratio 1/1/0.1), DOPE/MVL-5/DIR (1/0.2/0.1 and 1/1/0.1) and DOPC/DOTAP/DiR (1/1/0.1) were used to deliver eGFP-mRNA into CHO cells. The liposome-cell interactions of all DiR-formulations were analyzed by live cell microscopy using appropriate laser settings (Exc. 633 nm). For DOPE/DOTAP/DIR (mixing ratio 1/1/0.1) and DOPE/MVL-5/DIR (1/0.2/0.1) fusion signals were detected. However, within 3 minutes of incubation most efficient fusion was shown for DOPE/DOTAP/DIR (mixing ratio 1/1/0.1). For DOPE/MVL-5/DIR (1/1/0.1) and DOPC/DOTAP/DiR (1/1/0.1) only punctual DiR signals indicated membrane interactions of those formulations without membrane fusion.
